# Using Serenity Rooms and Similar Tools to Improve the Workplace during COVID-19: A Rapid Review

**DOI:** 10.3390/nursrep14010029

**Published:** 2024-02-05

**Authors:** Michael Mileski, Rebecca McClay, Clemens Scott Kruse, Joseph Baar Topinka, Katharine Heinemann, Brea Vargas

**Affiliations:** 1 School of Health Administration, Texas State University, San Marcos, TX 78666, USAjosephtopinka@txstate.edu (J.B.T.); katharineheinem@gmail.com (K.H.); breavargas@aol.com (B.V.); 2 School of Science, Technology, Engineering, and Math, American Public University System, Charles Town, WV 25414, USA; rebecca.mcclay@gmail.com

**Keywords:** serenity room, tranquility room, nurses, burnout, professional, burnout, psychological, retention, stress relief, well-being

## Abstract

This manuscript examines using serenity rooms and similar tools to improve the workplace during COVID-19 for nurses and other practitioners. A rapid review of the literature was conducted and completed from four different databases, including PubMed, CINAHL, Science Direct, and Academic Search Complete. The literature review was completed with the use of a single-string Boolean search to maximize the number of articles returned. The resulting 14 germane articles yielded six facilitator themes and four barrier themes. Facilitator themes included: benefits, assistive adjuncts, places of relaxation, leadership required, availability, and other effects. Barrier themes included: lacking leadership, concerns regarding lack of space, holistic concerns, and negative perceptions. There is a significant lack of research in the literature in this area. Most of the literature reviewed showed widely positive results for institutions that utilized serenity rooms or similar tools for decreasing nurse and practitioner stress and burnout. The use of these tools improved nurse and practitioner compassion, retention, and resiliency.

## 1. Introduction

### 1.1. Rationale

Bedside nurses today have an increased number of stressors to contend with in their workplace. The advent of COVID-19 compounded the effects of these stressors: the disease threatened nurses’ and other healthcare workers’ health, well-being, and ability to work [1]. Additionally, nurses documented extreme exhaustion, physical discomfort and injury from working conditions, fear of contracting COVID-19 themselves, and physical and emotional strain [1,2]. While the long-term effects of COVID-19 on the health and well-being of nurses are unknown, it is postulated that they will include cardiovascular diseases, physical manifestations, and mental health concerns [3,4]. Furthermore, approximately 80% of patients who had COVID-19 (nurses and healthcare staff included) may have one or more long-term sequelae from their own COVID-19 infection. This may lead to a decreased quality of life and impaired performance over time [5]. It is believed that these stressors have contributed to the turnover statistics of bedside nurses in the United States being 27.1% [6]. The average turnover cost for one bedside registered nurse (RN) ranges around USD 46,100, resulting in losses to the average hospital of USD 5.2–9.0 million per year [6]. Even a 1% decrease in the turnover rate would save the average hospital approximately USD 263,000 annually [6].

The idea of moral distress (situations in which an individual’s professional ethics are situationally constrained) and how it affected nurses and practitioners alike during the pandemic is of great concern. Nurses were required to work with acutely ill patients with a potentially fatal disease during a worldwide scarcity of personal protective equipment and other necessary resources [7]. This moral distress made practitioners feel they could not provide adequate care or care that aligned with their professional standards or ethics [7]. Many felt powerless during the pandemic to change the situation they faced, and many felt as if they were being judged by their peers, administration, and the families of their patients [7]. Moral distress is linked to healthcare providers being burned out and having intentions to leave their jobs in healthcare [7,8]. It is further postulated that moral distress results from modifiable unit characteristics, including a lack of adequate resources to meet healthcare providers’ personal needs or their needs to provide the appropriate quality of care [7,9]. Of further concern, moral distress issues have negative consequences on nurse and healthcare providers’ well-being, including post-traumatic stress disorder, burnout, and moral injury [10,11]. Relying on individual nurse ability or resilience is not the solution to dealing with personal trauma and burnout [10]. The employer’s response is critical in assisting both burnout and retention [10]. This further shows the need for sustainable solutions for nurses and healthcare providers to lessen these feelings and for the administration to find ways to adequately assist in diminishing them.

While the pandemic has subsided, and perhaps the related stressors have lessened, the focus on improving the workplace and supporting a healthy work environment has increased. Employer-driven models to decrease concerns among healthcare providers are more important now than ever.

### 1.2. Objectives

The purpose of this rapid review is to recognize underlying themes in using serenity rooms and similar tools to assist nurses and other healthcare staff by identifying the facilitators of and barriers to using these tools. This paper looks specifically at the utilization of serenity rooms and similar tools during the COVID-19 pandemic. It is hoped that this study will provide evidence-based rapid implementation initiatives for implementing these tools into hospitals and healthcare centers everywhere.

### 1.3. Population, Concept, and Context

Population: Important characteristics of the population focused upon for this study surrounded their being bedside or clinical healthcare practitioners, specifically nurses. Other populations were included due to a lack of relevant literature.

Concept: This rapid review looks specifically at using serenity rooms and similar tools to relieve stress and improve staff well-being.

Context: This rapid review focused specifically on hospitals; however, other healthcare environments were included as necessary due to a lack of relevant literature.

## 2. Materials and Methods

### 2.1. Overview

The research process began with a review of the intent of this manuscript by the researchers and associated healthcare terminology. To complete this review, PRISMA review standards [12], the Kruse protocol [13], and Arksey and O’Malley’s Scoping Studies Framework [14] were utilized. Four databases were queried to complete the literature review, including PubMed, CINAHL, Academic Search Complete, and Science Direct. A simple one-string Boolean search was utilized to maximize the number of articles returned from each database due to the small amount of works identified in the post-COVID-19 literature (2020 to present).

### 2.2. Inclusion Criteria

Study search terms and Boolean operators generated the database search string that resulted in the articles chosen to be included in the assessment for this manuscript. The Boolean string utilized was: (“serenity room”) OR (“quiet room”) OR (“tranquility room”) OR (“code lavender”) OR (“lavender lounge”). This generalized terminology was utilized after the researchers found that more detailed and specific strings excluded articles that may have potentially been included in the initial search for identification. The initial search resulted in 847 articles published between 1 January 2020 and September 2023. This date range was utilized to ensure that only COVID-19-related articles were being pulled related to the timeline in which they were published. COVID-19 was not included in the Boolean string because the inclusion of this terminology excluded germane articles. The researchers conducted the initial database query and identification of the article sample in early 2021. The article was tabled at that time due to a lack of literature. Subsequent database queries for this current manuscript began in July 2023, and the results and analyses were completed in September 2023.

### 2.3. Exclusion Criteria

Studies for this review were eligible if any mention of any of the terms surrounding the Boolean string chosen was present and if the article concerned any type of healthcare staff. Only high-quality journals that were peer-reviewed were utilized. Only articles published between 2020 and the present were selected for inclusion; any other articles were excluded. Finding germane works in the literature on the topic was a limiting factor in this critical topic.

**Figure 1:**
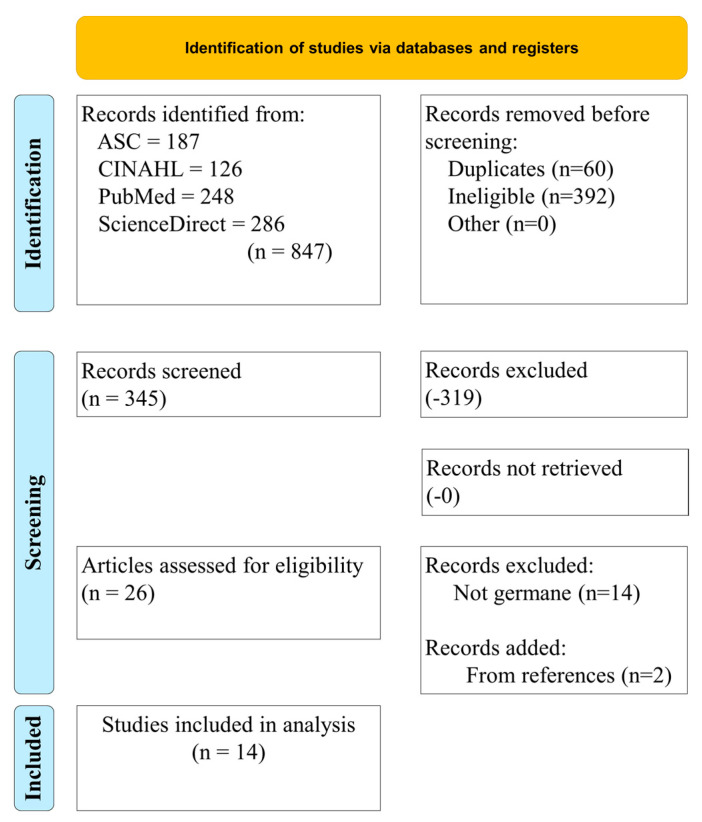
Preferred reporting items for rapid reviews and meta-analyses (PRISMA) diagram that demonstrates the study selection process.

Additional filters were included during the search process to produce focused and applicable results. Further, articles were only included in the rapid review if the researchers agreed upon them. At least three researchers had to agree to include each article. First, PubMed was queried fully, and the date range of 2020 to the present was used; non-germane articles were removed, resulting in six articles. Second, CINAHL was queried with PubMed excluded, which resulted in the removal of repeated articles and those that were not germane, resulting in zero articles. Academic Search Complete was then queried with the same criteria, which resulted in the removal of repeated articles and those that were not germane, resulting in zero articles. Lastly, Science Direct was queried with the same criteria, which resulted in the removal of repeated articles and those that were not germane, resulting in six articles. References from these 12 articles were assessed, and two additional articles were identified for inclusion, ultimately resulting in the 14 articles included in this rapid review. Figure 1 illustrates the rapid review process and the applied search exclusion criteria.

The authors conducted a rigorous review of the 14 articles chosen for inclusion in their analysis by reading the full manuscripts of each article. No less than three researchers read each article and agreed upon including each article in the study. No discrepancies were reported among the researchers while reviewing the articles for inclusion or dismissal. Article selection bias was addressed through a series of consensus meetings that focused on the articles chosen that were reviewed by the research team. No bias was identified, as all researchers fully agreed upon the articles included in the final review.

## 3. Results

### 3.1. Overview

Table 1 demonstrates the findings of the articles chosen for inclusion in this manuscript. Table 2 shows the coding of the studies and their strength and quality per the Johns Hopkins Nursing Evidence-Based Practice Model (JHNEBP). Based upon the results of these two tables, the researchers were able to pull facilitators and barriers from each of the papers regarding the use of serenity rooms for staff and to assign themes to these. Themes were decided upon by a consensus of the researchers and their review of the facilitators and barriers pulled from each of the included manuscripts. While the study design of each article would generally be an essential tenet of inclusion in this type of manuscript, it was not considered due to the small number of available studies for consideration.

The quality results of the identified studies, as assessed by the JHNEBP methodology, demonstrate that the majority of the articles (93%) came from level II (50%; quasi-experimental studies) and level III (43%; non-experimental or qualitative studies) categories. Only one article came from the level IV (7%; opinion based on clinical evidence) category. While the strength of most of the studies utilized in this paper is of note, it is important to realize that there is a significant lack of knowledge in the currently available publications despite widespread knowledge surrounding the use of serenity rooms and associated tools. Due to the lack of available literature, the researchers attempted to utilize any available literature, regardless of its quality or where it came from worldwide.

The rapid review process identified articles that were published after the COVID-19 pandemic that were related to the implementation or use of serenity rooms or other similar concepts. Of the total sample of articles (*n* = 14), each researcher read the assigned articles in the sample and worked to establish a consensus on the major themes for the facilitators and barriers extracted from each article. From this extraction, affinity matrices were constructed for both facilitators and barriers. Each affinity matrix (presented below in Table 3 and Table 4) shows a percentage occurrence of each theme compared to the total sample. For the purposes of this paper, the terms serenity room, serenity space, restorative space, quiet room, respite space, respite area, tranquility room, lavender lounge, Team Lavender, and Code Lavender are used synonymously to refer to the spaces that are being studied. These terms change depending on the publications within which they are found.

### 3.2. Facilitators

Six facilitator themes were identified. Their occurrence, frequency sum, and percent frequency are shown in Table 3. The positive facilitator theme “benefits” was mentioned in 15 of 37 occurrences, or 40.54% [15,16,17,18,19,20,21,22,23,24,25,26]. Quiet rooms increased compassion satisfaction among staff members and decreased burnout and secondary stress in nurses [15]. The interventions used to improve the working environment positively impacted nursing staff, leading to improved resiliency and retention [16]. Staff members who used massage chairs, which were part of the serenity rooms, had significantly lower feelings of anxiety [17]. There were overall findings stating that restorative spaces were able to improve clinician well-being and resilience levels [18]. Advanced practice registered nurses (APRNs) found that quiet rooms were an assistive tool in preventing burnout (16.2% strongly agreed and 54.5% agreed out of those surveyed) [19]. Serenity spaces were beneficial when provided as a quiet space with music and pictures; this allowed staff to take a break from patients, relax, and meditate [20]. When management was involved with the use of evidence-based approaches, tools such as lavender rooms or Code Lavender were shown to soothe the human spirit and enhance staff well-being [21]. Code Lavender and lavender lounges have emerged as a strategy to provide a stress-defusing environment for staff [22]. Further, these spaces offer a space for staff to retreat to for relaxation, meditation, and uninterrupted reflection [22]. The ability of the staff to “tap out” and have a serenity break allows them to have a time out, allowing for improved mental well-being and renewed practice abilities [23]. Serenity rooms were beneficial as an accessible retreat for staff who had difficulty coping with the atrocities of the pandemic [23]. Serenity rooms provided a vehicle for self-care for staff to emotionally recharge away from their workspace in order to prevent burnout [23]. When Code Lavender protocols are in place, they allow for the resources, time, and activities necessary for staff to be able separate themselves from the workplace when critical situations occur [24]. Formal and informal implementations of Code Lavender protocols have proven to be excellent in assisting with emotional, spiritual, and psychological health and well-being [25]. Moreover, 74% of those using lavender lounges reported that their use was somewhat, moderately, or very helpful in reducing stress [26].

The facilitator theme “assistive adjuncts” was mentioned in 8 of the 37 occurrences, or 21.62% [15,17,18,19,27]. Specific colors on the room’s walls, massage chairs, and aromatherapy can decrease stress and facilitate relaxation [15]. The use of massage chairs reduced feelings of emotional exhaustion, burnout, frustration, being worn out, stress, and anxiety [17]. Furthermore, the longer the massage chair was used, the lower the emotional exhaustion and anxiety levels became [17]. Overall, having a massage chair in the room was an effective part of a holistic strategy to care for frontline nurses [17]. Lighting, other décor, and essential oils can contribute to positive outcomes for relaxation and care [17]. Other assistive tools such as diffusers, light dimmers, blankets, pillows, sound machines, integrative therapies, mindfulness exercises, virtual reality, music therapy, yoga, adult coloring books, Watson Caritas cards, soundproof walls, no-talk zones, no-electronics zones, therapy animals, chaplains, social workers, and behavioral health support can be excellent tools to assist staff towards relaxation and decreasing stress [18]. With the use of fresh paint and comfortable furniture, unused patient rooms can quickly be transformed into quiet rooms [19]. Chaplains can also serve as a valuable adjunct to Code Lavender and other support protocols to support staff when they are distressed after traumatic or stressful events [27].

The facilitator theme “places of relaxation” was mentioned in 7 of the 37 occurrences, or 18.92% [15,16,18,25,28]. Nurses can use quiet rooms as a place of relaxation [15]. After using serenity rooms, 71.1% of staff members felt renewed [16]. Serenity rooms were felt to be renewing and relaxing while providing a place of privacy for staff [16]. It was found that ample daylight and separation from work areas are necessary requirements for a respite space to be successful for staff [18]. When nurses “call” a Code Lavender, another nurse can step in for them for fifteen minutes with no questions asked [18]. Staff members show appreciation for the availability of tranquility rooms as a break from the hustle and bustle of their workplace [28]. Serenity spaces created during the pandemic were important so that staff had time away from patients’ bedsides; they provided positive results for staff who used them [25].

The facilitator theme “leadership required” was mentioned in 5 of the 37 occurrences, or 13.51% [18,25,26,28]. Support for any of these models must come from team leaders, along with a caring culture within the workplace [18]. Using the serenity room can become problematic when team leaders in the workplace do not place adequate importance on their use [28]. Managers must focus on the availability of these rooms and allow staff to use them instead of focusing on the workplace and the rush of what is happening around them [28]. Proper use of the Team Lavender approach moves the workplace from a “keep your chin up and carry on” environment to one that is assistive to healthcare workers’ health, safety, and wellness instead [25]. Finding facilitators for using lavender lounges includes providing coverage by colleagues, visiting during lunch breaks, and having low unit acuity, all of which require the assistance of team leaders [26].

Two additional facilitator themes were identified to include “availability” [18] and “other effects”, [16] each of which was mentioned in 1 of the 37 occurrences, or 2.7% each. For any of these assistive spaces to be effective, they must be available to staff on a 24/7 basis [18]. When staff that used these assistive spaces were surveyed, 94% believed that other departments would benefit from having their own serenity spaces to take advantage of [16].

### 3.3. Barriers

Six barrier themes were identified. Their occurrence, frequency sum, and percent frequency are shown in Table 4. The negative barrier theme “lacking leadership” was mentioned in 12 of 15 occurrences, or 80% [16,18,26,28]. Staff were concerned that limited resources, including staffing, time, and room size, could negatively affect their ability to take advantage of the serenity room [16]. Several studies cited staff concerns about not having enough time to use the serenity rooms [16,26,28]. Limited staffing being provided on the floor prevented staff from being able to use the rooms [16,26]. Simply having these beneficial rooms available is not enough [26,28]. Staff members listed room sizes or lack of space as a concern [16,18]. Managers must support staff members in the use of these spaces. When managers do not support staff to escape to these spaces when needed, it can lead to increased stress and decreased mental health and well-being [16,26]. Staff also need to feel supported by their leaders in using these serenity rooms, as many feel uncomfortable using them or believe that perceptions of others towards them may change because they have utilized these rooms and their leaders are not supporting them [16,26]. Other barriers to using these spaces include high unit acuity, high unit census, and high patient care demands [26].

Three additional barrier themes were identified to include “concerns regarding lack of space” [18], “holistic concerns” [17], and “negative perceptions” [26], each of which was mentioned in 1 of 15 occurrences, or 6.67% each. There is an overall belief that a lack of respite areas can affect nurse resilience and well-being [18]. There are opinions regarding specific lighting choices, décor, and essential oils contributing negatively to these respite areas [17]. Some nurses may choose not to use lavender lounges due to their perceptions of what others may think about them [26].

## 4. Discussion

### 4.1. Implementation and Process Improvement

Based on the findings of this rapid review, when put into place by hospital managers, serenity rooms have significant benefits for practitioners and administration alike. Serenity rooms are understood to prevent burnout in nurses and practitioners. The ability of staff to have a break away from the healthcare environment where they fully unplug from patients, families, and the rigors of what they are experiencing on the floor is an integral part of decreasing stress and increasing the practice ability of practitioners.

### 4.2. Improving Nurses’ Well-Being

The use of serenity rooms can provide a myriad of benefits for nurses. First, stepping away from the rigors of their workplace allows nurses to disengage for a few moments, thus improving their compassion satisfaction when they return. Using a serenity room is understood to decrease burnout, secondary stress, anxiety, emotional exhaustion, and feelings of being worn out. They are also understood to increase emotional, spiritual, and psychological well-being and health. Workplaces that established these serenity rooms during the pandemic allowed their staff a place of respite away from the atrocities of the pandemic. Any, or all, of these things can have significant effects on nurse and practitioner job satisfaction and retention.

### 4.3. Retention and Resiliency

The use of serenity rooms clearly benefits employers via increased practitioner retention and resiliency. The rooms put into place by employers allow staff a place to diffuse their stress and emotionally re-energize themselves.

### 4.4. Management Buy-In

The involvement of team leaders/management seems to be a critical factor in the success of the implementation of serenity rooms. Facilities with these spaces enjoyed staff with renewed feelings of well-being and a noted soothing of the human spirit for staff who utilized them. However, team leaders must be a part of these concepts, as establishing a space for the serenity room to occupy is only one aspect of its success. Further, managers must allow nurses to leave the floor to take advantage of these essential tools to bolster their well-being. Lack of staff, high acuity, and other concerns must not get in the way of staff taking a few moments of respite to utilize these rooms to recharge themselves. Allowing nurses time away from patients’ bedsides is vital to successfully implementing these spaces.

## 5. Study Limitations

This study is limited by the small amount of research that has been conducted on the topic. Most of the research was published in acceptable journals with acceptable JHNEBP scoring. Much gray literature on this topic also exists, which is opinion-based at best. Further research showing the benefits to nurses, practitioners, and managers could be leveraged to develop and use these essential tools for employee health and well-being.

## Figures and Tables

**Table 1:** Summary of findings (*n* = 14).

Year	Author	Facilitator	Theme	Barrier	Theme
2019	Hu [15]	Quiet room can be used by nurses as a place to relax.	Places of relaxation	Lack of quiet room use led to decreased compassion satisfaction and increased burnout and secondary stress in nurses.	Lacking leadership
		Colors on the walls of the room, massage chairs, and aromatherapy diffusers can decrease stress and facilitate relaxation.	Assistive adjuncts		
		Quiet rooms can increase compassion satisfaction and decrease burnout and secondary stress in nurses.	Benefits		
2020	Salmela et al. [16]	In total, 71.1% of staff surveyed felt renewed after using the serenity room.	Places of relaxation	Staff who did not use the serenity room cited limited staffing or not enough time in their shift to utilize the room.	Lacking leadership
		Almost all (94%) of the staff surveyed thought that other departments would benefit from having their own serenity room.	Other effects	Staff were concerned about limited resources, including staffing, time, and room size, impacting their ability to use the serenity room.	Lacking leadership
		Serenity rooms were felt to be renewing and relaxing, while providing a place of privacy.	Places of relaxation	Staff need to be supported to use the serenity room, and when there are no other staff to relieve them it became difficult to leave their workstations (especially for doctors and advanced practice nurses).	Lacking leadership
		Interventions to improve hospital working environments positively impact nursing staff, which may lead to improved resiliency and retention.	Benefits	Staff need to feel comfortable enough to use serenity rooms, and this requires the support of coworkers and managers.	Lacking leadership
2022	Pagador et al. [17]	The use of massage chairs decreased feelings of emotional exhaustion, burnout, frustration, being worn out, stress, and anxiety.	Assistive adjuncts	Lighting, other décor, and essential oils may have contributed to negative outcomes and further examination of the comprehensive holistic environment is necessary.	Holistic concerns
		Longer use of massage chairs equated to lower levels of emotional exhaustion and anxiety.	Assistive adjuncts		
		Feelings of anxiety were significantly lower in those who used the massage chair for longer than 20 min compared to those who used it for 10 min.	Benefits		
		Massage chairs in serenity room are effective as part of a holistic strategy to care for frontline nurses’ well-being.	Assistive adjuncts		
		Lighting, other décor, and essential oils may have contributed to positive outcomes.	Assistive adjuncts		
2022	Gregory et al. [18]	Restorative spaces improve clinician well-being and resilience.	Benefits	Lack of respite areas affects nurses’ resilience and well-being.	Concerns regarding lack of space
		Ample daylight and separation from work areas are necessary parts of a successful respite space for staff.	Places of relaxation	Lack of space is often blamed for the lack of respite areas for staff members.	Lacking leadership
		Multiple tools can be built into restorative spaces successfully, which include: diffusers, light dimmers, blankets, massage chairs, pillows, sound machines, integrative therapies, mindfulness exercises, virtual reality, music therapy, yoga, adult coloring books, Watson Caritas cards, soundproof walls, no-talk zones, no-electronics zones, therapy animals, chaplains, social workers, behavioral health support, and others.	Assistive adjuncts		
		Support for these models must come from team leaders and have a caring culture to back it.	Leadership required		
		To be successful, these restorative spaces and tools need to be available 24/7.	Availability		
		Calling a “Code Lavender” allows nurses to step away for 15 min while another nurse steps in for them with no questions asked.	Places of relaxation		
2021	Stallter and Gustin [19]	A quiet room for APRNs was an assistive tool for preventing burnout: 54.5% agreed with this, and 16.2% strongly agreed, in those surveyed.	Benefits		
		Unused patient rooms with adequate ventilation can be used as quiet rooms by adding fresh paint and comfortable furniture.	Assistive adjuncts		
2022	Vranas et al. [20]	Serenity spaces were created during the pandemic which allowed staff to be away from patients’ bedsides with positive results.	Places of relaxation		
		A special quiet break room was provided with music and pictures which allowed staff to take a break and relax away from their patients. The spaces also allowed for meditation.	Benefits		
2022	Carter and Bogue [21]	Management support for evidence-based approaches, such as Code Lavender, can soothe the human spirit and enhance staff well-being.	Benefits		
2022	White et al. [22]	Code Lavender and lavender lounges have emerged as a strategy to provide a stress-defusing environment for staff.	Benefits		
		Lavender rooms or lounges provide a space for staff to retreat for relaxation and meditation. They allow a quiet place to reflect and relax uninterrupted.	Benefits		
2021	Coicou [23]	The ability of the staff to be able to “tap out” and have a serenity break can make the difference in the success of a nurse’s mental well-being and practice ability.	Benefits		
		Serenity rooms acted as an accessible retreat for staff who were having difficulty in coping with the atrocities of the pandemic.	Benefits		
		Serenity rooms allow for self-care stations for staff to emotionally recharge away from their work area in order to prevent burnout.	Benefits		
2021	Kelly et al. [24]	Having Code Lavender processes in place allows for the resources, time, and activities of staff to be covered when critical situations occur.	Benefits		
2022	Orton [25]	The availability of a team Code Lavender approach (“Team Lavender”) debunks the “keep your chin up and carry on” ideology that has persisted for years and can be detrimental to the health, safety, and wellness of healthcare workers.	Leadership required		
2023	Smith et al. [26]	Facilitators of the use of lavender lounges include coverage by colleagues, visiting during lunch breaks, and having low unit acuity.	Leadership required	Barriers to use of the lavender lounge include high unit acuity, high unit census, and high patient care demands.	Lacking leadership
		A total of 74% of those using the lavender lounge reported it was somewhat, moderately, or very helpful in reducing their stress.	Benefits	Nurses may choose to not use the lounge due to their perceptions of what others might think of them.	Negative perceptions
				Some nurses were unable to use the lavender lounge because it took too much time away from their clinical responsibilities in the ICU. This shows an impracticality of the lavender lounge for this population and a concern for nurses’ health and well-being.	Lacking leadership
				Simply having a room has no benefits at all: the nurses must be allowed to take advantage of the available resources.	Lacking leadership
2022	Tartaglia et al. [27]	Chaplains can be used as an adjunct to Code Lavender and other support protocols to provide support to staff in distress following traumatic or stressful events.	Assistive adjuncts		
2023	Haugland et al. [28]	Staff members appreciate the availability of tranquility rooms as a break from the hustle and bustle of the workplace.	Places of relaxation	Staff believe that tranquility rooms are useless if there is no time allotted to use them.	Lacking leadership
		Workplaces need to focus on allowing staff to be able to use serenity rooms instead of focusing only on the rush of the workplace.	Leadership required	Staff do not necessarily have time to use serenity rooms.	Lacking leadership
		Staff would like more time in the serenity room to get away from the workplace.	Leadership required	Simply providing a serenity room and similar tools is not enough for them to be successful. Managers must focus on the mental health and well-being of their staff members.	Lacking leadership

**Table 2:** Johns Hopkins Nursing Evidence-Based Practice Model ratings for each article.

Year	Author	Strength	Quality
2019	Hu [15]	IV	B
2020	Salmela et al. [16]	II	B
2022	Pagador et al. [17]	II	B
2022	Gregory et al. [18]	III	B
2021	Stallter & Gustin [19]	II	A
2022	Vranas et al. [20]	II	A
2022	Carter & Bogue [21]	III	B
2022	White et al. [22]	III	B
2021	Coicou [23]	III	B
2021	Kelly et al. [24]	III	B
2022	Orton [25]	III	B
2023	Smith et al. [26]	II	A
2022	Tartaglia et al. [27]	II	B
2023	Haugland et al. [28]	II	A

**Table 3:** Facilitator themes.

Facilitators	Occurrences	Sum	%
Benefits	15–26	15	40.54
Assistive adjuncts	15, 17–19, 27	8	21.62
Places of relaxation	15, 16, 18, 25, 28	7	18.92
Leadership required	18, 25, 26, 28	5	13.51
Availability	18	1	2.70
Other effects	16	1	2.70
	TOTAL	37	

**Table 4:** Barrier themes.

Barriers	Occurrences	Sum	%
Lacking leadership	16, 18, 26, 28	12	80
Concerns regarding lack of space	18	1	6.67
Holistic concerns	17	1	6.67
Negative perceptions	26	1	6.67
	TOTAL	15	

## Data Availability

The data presented in this study are available in the articles listed in references.

## Public Involvement Statement

No public involvement was included in any aspect of this research.

## Guidelines and Standards Statement

This manuscript was drafted according to the PRISMA review standards [12], the Kruse protocol [13], and Arksey and O’Malley’s Scoping Studies Framework [14].
